# Early Disruption of Cortical Sleep-Related Oscillations in a Mouse Model of Dementia With Lewy Bodies (DLB) Expressing Human Mutant (A30P) Alpha-Synuclein

**DOI:** 10.3389/fnins.2020.579867

**Published:** 2020-09-17

**Authors:** Myrto Stylianou, Boubker Zaaimi, Alan Thomas, John-Paul Taylor, Fiona E. N. LeBeau

**Affiliations:** ^1^Biosciences Institute, Faculty of Medical Sciences, Newcastle University, Newcastle upon Tyne, United Kingdom; ^2^School of Life & Health Sciences, Aston University, Birmingham, United Kingdom; ^3^Translational and Clinical Research Institute, Faculty of Medical Sciences, Newcastle University, Newcastle upon Tyne, United Kingdom

**Keywords:** slow oscillations, prefrontal cortex, hippocampus, fast network oscillations, alpha-synuclein, spindles

## Abstract

Changes in sleep behavior and sleep-related cortical activity have been reported in conditions associated with abnormal alpha-synuclein (α-syn) expression, in particular Parkinson’s disease (PD) and dementia with Lewy bodies (DLB). Notably, changes can occur in patients years before the onset of cognitive decline. Sleep-related network oscillations play a key role in memory function, but how abnormal α-syn impacts the generation of such activity is currently unclear. To determine whether early changes in sleep-related network activity could also be observed, prior to any previously reported cognitive dysfunction, we used mice that over-express human mutant α-syn (A30P). Recordings *in vivo* were performed under urethane anesthesia in the medial prefrontal cortex (mPFC) and CA1 region of the hippocampus in young male (2.5 – 4 months old) A30P and age-matched wild type (WT) mice. We found that the slow oscillation (SO) < 1 Hz frequency was significantly faster in both the mPFC and hippocampus in A30P mice, and Up-state-associated fast oscillations at beta (20 – 30 Hz) and gamma (30 – 80 Hz) frequencies were delayed relative to the onset of the Up-state. Spindle (8 – 15 Hz) activity in the mPFC was also altered in A30P mice, as spindles were shorter in duration and had reduced density compared to WT. These changes demonstrate that dysregulation of sleep-related oscillations occurs in young A30P mice long before the onset of cognitive dysfunction. Our data suggest that, as seen in patients, changes in sleep-related oscillations are an early consequence of abnormal α-syn aggregation in A30P mice.

## Introduction

Dementia with Lewy bodies (DLB) is the second most common cause of dementia after Alzheimer’s disease (AD) (Walker et al., 2015). Clinically DLB is characterized by dementia, cognitive fluctuations, rapid eye movement (REM) sleep behavioral disorder (RBD), parkinsonism and visual hallucinations (McKeith et al., 2017). RBD is a condition where the mechanisms causing muscle atonia during REM sleep are lost, resulting in the enactment of dream behaviors. RBD is the best clinical predictor of a set of diseases collectively called α-synucleinopathies, which includes DLB, Parkinson’s disease (PD) and multiple system atrophy (MSA), occurring years before the onset of dementia and Parkinsonism (McKeith et al., 2020). However, other DLB-related sleep problems have been identified including sleep fragmentation, poor sleep quality, insomnia, increased sleep onset latency, more time spent in lighter sleep and vivid dreams (Terzaghi et al., 2013; Chwiszczuk et al., 2016; Fernández-Arcos et al., 2019).

Sleep-related network oscillations can be recorded using the electroencephalogram (EEG) in both humans and rodents during awake and sleep states. Sleep consists of two distinct patterns of cortical activity, REM sleep and non-REM (NREM) sleep that can be readily distinguished in local field potential (LFP) recordings. REM sleep is defined by low amplitude, oscillatory activity predominantly in the theta (4 – 8 Hz) band. In contrast, NREM sleep is characterized by a SO < 1 Hz and sleep spindles at 8 – 15 Hz (Volgushev et al., 2006; Ruiz-Mejias et al., 2011). Both REM and NREM sleep play a central role in memory consolidation and synaptic plasticity (Timofeev and Chauvette, 2017; Klinzing et al., 2019; Puentes-Mestril et al., 2019).

In rodents the SO can be recorded during both natural sleep (Sirota et al., 2003; Kim et al., 2015), and under urethane or ketamine anesthesia (Steriade et al., 1993a; Clement et al., 2008; Ruiz-Mejias et al., 2011; Pagliardini et al., 2013a; Barthó et al., 2014). The SO is present throughout the neocortex (Ruiz-Mejias et al., 2011), and is also observed in the hippocampus (Wolansky et al., 2006; Sharma et al., 2010). The regular fluctuations in the LFP SO activity reflect regular cycles of depolarization (Up-state) and hyperpolarization (Down-state) of cortical pyramidal cells (Steriade et al., 1993b). During the Up-state excitatory and inhibitory synaptic inputs occur which occasionally reach threshold for action potential firing (Haider et al., 2006). Fast network activity, notably in the beta and gamma frequency ranges, occur on the Up-state in cortex (Steriade et al., 1996; Compte et al., 2008) and hippocampus (Wolansky et al., 2006).

In addition, spindle activity between 8 and 15 Hz also occurs on the Up-state (Barthó et al., 2014; Kim et al., 2015). Changes in sleep spindles have been reported in PD (Christensen et al., 2014; Latreille et al., 2015) and DLB (Fernández-Arcos et al., 2019). Furthermore, at least in PD patients, reduced spindle density reliably predicted the subsequent progression to dementia (Latreille et al., 2015). Hippocampal sharp wave ripples (SPW-R) are characterized by a slow field potential sharp wave (SPW) in the CA1 region of the hippocampus with a faster (∼80 – 200 Hz) ripple nested on the slow wave (Buzsáki, 2015). SPW-R in the hippocampus are temporally coherent with cortical spindles in both rodents (Siapas and Wilson, 1998) and humans (Clemens et al., 2011; Staresina et al., 2015).

To-date the majority of EEG studies in patients with DLB have been conducted during the awake state where a well described ‘slowing’ of the dominant frequency in the EEG is observed (Briel et al., 1999; Bonanni et al., 2016; Stylianou et al., 2018). Using mice that over-express human wild-type α-syn to model DLB, a shift of power from higher alpha to lower theta frequencies was also found in awake mice (Morris et al., 2015), and during NREM and REM sleep (McDowell et al., 2014). Studies in transgenic α-syn mice which have also shown alterations in the sleep-wake cycle in awake-behaving animals (Rothman et al., 2013; McDowell et al., 2014; Peters et al., 2020). However, detailed studies of the SO and spindles in murine models of α-syn are currently lacking. There is now considerable interest in prodromal DLB/PD symptoms which include RBD (McKeith et al., 2020) and changes in sleep spindles (Latreille et al., 2015). Therefore, we wished to determine whether mice expressing the mutant (A30P) human α-syn (Kahle et al., 2000; Neumann et al., 2002; Schell et al., 2009) might, as seen in human studies, exhibit changes in sleep-related oscillations prior to the onset of any previously reported cognitive and motor symptoms. Human mutant α-syn is upregulated from 1 month of age in A30P mice (Kahle et al., 2000). Subsequently there is an age-dependent increase in α-syn pathology including oligomerization, fibrillization and increased phosphorylation at serine 129 (P129) in A30P mice (Schell et al., 2009), resulting in cognitive dysfunction at 12 months of age (Freichel et al., 2007; Schell et al., 2009). In particular, A30P mice exhibit impaired fear conditioning, impaired performance in the Morris water maze and reduced active avoidance compared to control mice at 12 months, but not at 4 months of age (Freichel et al., 2007). Major motor dysfunction, including rigidity and tremor, follows the cognitive dysfunction at ∼14 months of age (Kahle et al., 2000; Keane et al., 2019). Therefore, to determine whether changes in sleep-related oscillations occurred before any of the reported cognitive dysfunctions in A30P mice we used animals at 2.5 – 4 months of age.

Recordings were made under urethane anesthesia, a widely used model of NREM sleep in rodents (Steriade et al., 1993a; Barthó et al., 2014). We assessed several features, including SO frequency, power on the Up-state, neuronal firing rates and spindle activity in A30P mice compared to WT mice. In view of the known functional and anatomical differences between mPFC subregions (Kesner and Churchwell, 2011), we have recorded LFP and single neuron activity from anterior cingulate cortex (ACC), prelimbic (PrL), infralimbic (IL), and dorsal peduncular region (DP) of the mPFC and only the LFP in CA1 of hippocampus. We identified several changes including, a faster SO frequency in all subregions in mPFC and also in hippocampus, and increased neuronal firing on the Down-state and impaired spindle activity in the mPFC of A30P mice. We demonstrated that changes in NREM sleep-related network activity in A30P mice occur long before the onset of measurable cognitive dysfunction.

## Materials and Methods

### Animals

A30P mice (male) expressing human mutant A30P α-syn under the control of the Thy-1 promoter (Kahle et al., 2000) were used. The A30P line was regenerated from frozen sperm from breeding pairs originally supplied by Dr. P. Kahle, University of Tubingen. Sperm was used to inseminate a wild type (WT) C57B/6 female to generate a heterozygous line. The pups were genotyped (Transnetyx, Cordova, TN, United States) to ultimately obtain homozygous A30P and wild-type (WT) lines. These lines were bred in-house but were frequently regenerated by outbreeding to WT females and further genotyping.

All procedures described below were in accordance with the UK Animals (Scientific Procedures) Act 1986 and the European Union Directive 2010/63/EU. WT and A30P mice were housed at Newcastle University’s animal facility in a temperature- and humidity-controlled environment consistent with the ARRIVE (Animal Research: Reporting of *In Vivo* Experiments) guidelines. Mice were kept in an enriched environment (cage toys) under a 12 h light:dark cycle (lights on 7 am–7 pm) with access to food and water *ad libitum*. Mice were group housed and experiments were commenced ∼2 h into the light (sleep) phase of the circadian cycle.

### Anesthesia and Surgery

The animals were anesthetized with 4% isoflurane followed by urethane (0.6 ml of 20 g/kg i.p; Sigma-Aldrich). Mice were then transferred to a stereotaxic frame (Kopf, Tujunga, CA, United States) placed on a heating pad with feedback temperature control via a rectal probe (Harvard Apparatus, Holliston, MA, United States) with core temperature maintained at 36.8°C. The mice breathed spontaneously but to maintain an oxygen saturation of >95%, medical oxygen (BOC Industrial Gases, United Kingdom) was supplied through a tube mounted to the nose bar of the stereotaxic frame. For the duration of the surgery (20–30 min), 1% isoflurane was also provided through the mask. The isoflurane concentration was then gradually reduced and stopped when a deep plane of urethane anesthesia was achieved. The level of anesthesia was confirmed by absence of the pedal withdrawal reflex. The reflex was checked regularly and additional doses of urethane (3.3 g/kg) were given i.p. if indicated.

A skin incision was made in the scalp and infused with lidocaine, before the periosteum was retracted to expose bregma. A craniotomy was drilled above the left mPFC and/or the right hippocampus. Electrodes (see below) were implanted into the left mPFC (AP = + 2, ML = −0.4, DV = + 2.5 mm) and/or right hippocampus passing through the CA1 region of the hippocampus (AP = −2, ML = + 2, DV = + 2 mm, at 7.8° angle). The electrode was lowered ventrally through the dura using a one-axis oil-filled hydraulic micromanipulator (Narishige, Japan).

### Data Recording and Acquisition

**FIGURE 1:**
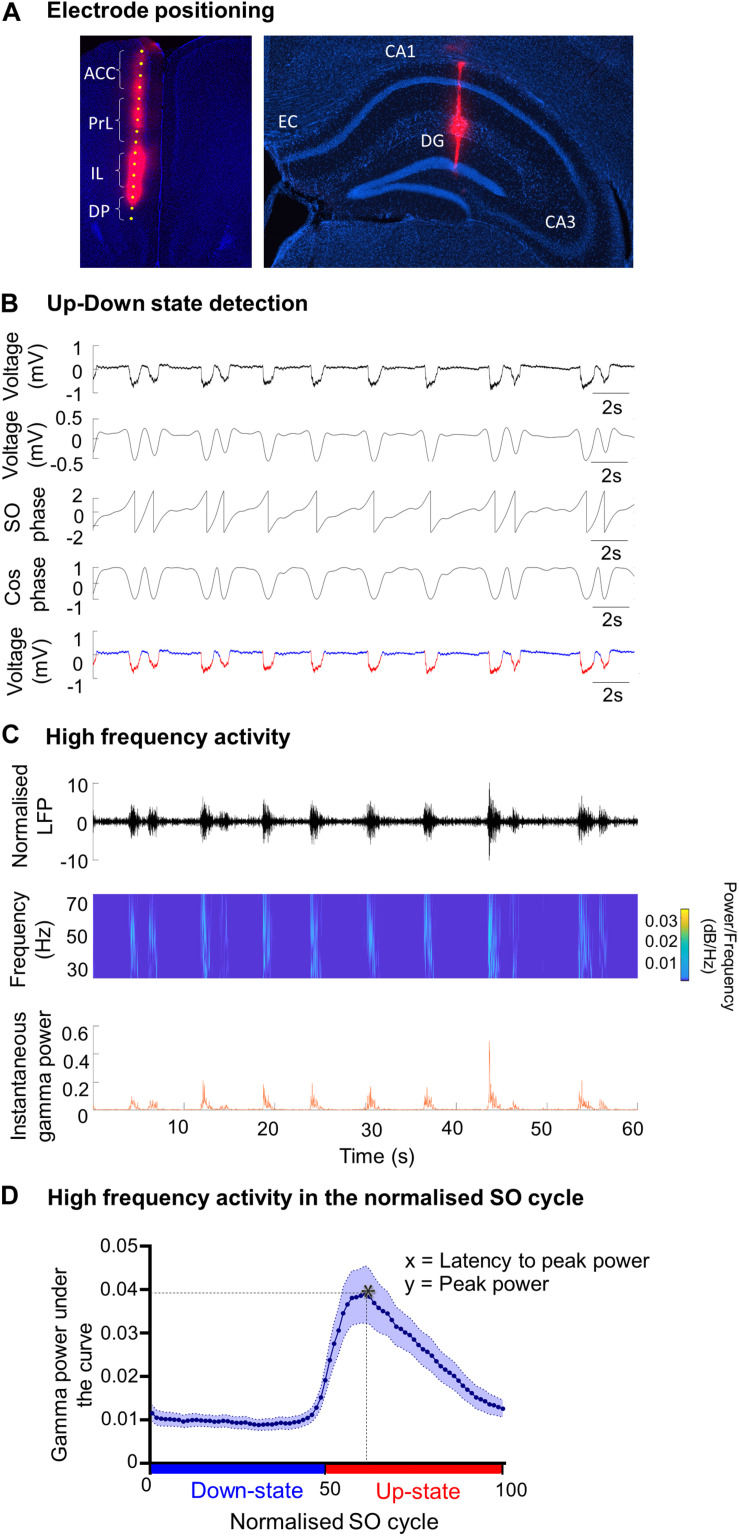
Analysis methods of SO characteristics and the associated fast oscillations. **(A)** Example of DiI staining showing the electrode tract in (i) the medial prefrontal cortex (mPFC) and its four subregions: anterior cingulate cortex (ACC), prelimbic cortex (PrL), infralimbic cortex (IL) and dorsal peduncular (DP), and (ii) in the hippocampus passing though the CA1, visualized using DAPI staining. EC = entorhinal cortex; DG = dentate gyrus. **(B)** Up- and Down-state detection method. The LFP was band-passed for the SO (0.1–0.9 Hz) and a Hilbert transform was used to calculate the phase of the SO. The threshold to discriminate between Up- and Down-states was cos(ϕ(t) = 0). The mean Up- and Down state-lengths were calculated for each channel, for each recording. Red = Up-state; blue = Down-state. **(C)** Example of power extraction in the gamma band in the mPFC. The LFP data for each electrode channel was normalized using the *z*-score and was then transformed using a Morlet wavelet for that frequency band (here gamma: 30–79.9 Hz). The instantaneous area power was calculated using trapezoidal numerical integration over all frequencies in the given band. **(D)** The average gamma band power distribution in the SO cycle across one recording. Each SO cycle (Down-state + Up-state) was normalized (0 = start of Down-state, 50 = Down to Up-state transition, 100 = end of Up-state) and the area under the curve (power) was calculated for each point in this cycle, for each frequency range. The high frequency power at each point was averaged across the SO cycle (blue curve), allowing the extraction of the average power on the Up- and Down-state, and of the peak power and its latency, in the SO cycle for each frequency band. * indicates peak power and latency.

Multi-channel recordings were made in all subregions of the mPFC in the left hemispheres and/or the contralateral hippocampus passing through the CA1 (Figure 1A). Single shank 16-channel silicon probes (150 μm inter-site spacing; 25 μm diameter, Atlas Neuroengineering, Leuven, Belgium) were used. Before insertion, the silicon probe was coated with a fluorescent dye (DiI) (1,1′-dioctadecyl-3,3,3′,3′-tetramethylindo-carbocyanine; Molecular probes, Eugene, OR, United States), dissolved in DMSO (1.5–2.5 mg/ml). The signals were passed through a unity-gain headstage (Plexon, TX, United States), and amplified (×1000) and filtered (0.07–300 Hz for LFP) by a Plexon preamplifier (Plexon, TX, United States). The LFP was digitized at 1 kHz, while neuronal spike data were digitized at 40 kHz and recorded using Plexon software (Sort Client).

### Histological Verification of Recording Site Position

After the experiment the mouse was sacrificed by injection with Euthatal (0.2 ml i.p.). The brain was removed from the skull and post-fixed in 4% paraformaldehyde (PFA) and 0.1 M phosphate buffer solution (PBS) at 4°C, for approximately 48 h. The brain was then transferred for cryoprotection in a 30% sucrose solution for 48–72 h. Coronal sections (60 μm) were cut on a cooled vibratome (Zeiss Hyrax V50, Zeiss, Oberkochen, Germany) and collected in 0.1 M PBS. DAPI (4′,6-diamidino-2-phenylindole; Sigma-Aldrich) staining was used to verify the position of single channel tungsten electrodes, and DiI (D282, Thermo Fisher Scientific) was used to verify the position of the silicon probes (Figure 1A). After staining, sections were mounted and cover-slipped using mounting medium (Sigma-Aldrich). The location of the electrodes was verified using the Fiji plugin on ImageJ (Schindelin et al., 2012).

### Data Analysis

#### Data Pre-processing

The raw LFP was band-stop filtered at 50 Hz. The LFP recordings used were concatenated sections from 30 min-long recordings taken once SO activity was clearly established. The resulting LFP recordings of SO were approximately 20 min-long. All data analysis was performed offline using custom MATLAB (Mathworks, Natick, MA, United States) scripts.

#### Up-State and Down-State Detection

Up- and Down-state (UDS) detection (Figure 1B) was performed using the phase of the SO, as described previously (Massi et al., 2012), except that the Hilbert transform (rather than the wavelet transform) was used to calculate the phase of the SO (Gretenkord et al., 2017). The LFP was first band-pass filtered for the SO (0.1–0.9 Hz) and the instantaneous phase ϕ(t) was calculated using the Hilbert transform. The threshold to discriminate between Up- and Down-states was cos(ϕ(*t*) = 0) (Figure 1B). To qualify as an Up-state, the duration of the event had to exceed 300 ms and the average amplitude of the Up-state of the SO band-passed signal had to be larger than 0.05 mV. The mean Up- and Down-state lengths were calculated for each channel, for each recording. The SO cycle length was calculated as the length of the Down-state + the length of the following Up-state. The number of SO cycles was used to calculate the frequency of the SO. The amplitude of the SO was calculated using the SO-filtered LFP (0.1–0.9 Hz). The SO amplitude was calculated as the peak-to-trough amplitude (maximum during Up state–minimum during Down state) for each cycle.

#### Calculation of the UDS Phase Vector

Time points of Up- and Down-state transitions were calculated from the UDS detection logical vector. A cycle always consisted of a Down-state and an Up-state and contained three transitions: transition 1: Up-to-Down transition (i.e., end of the Up-state to beginning of the Down-state) 2: Down-to-Up transition (i.e., end of the Down-state to beginning of the Up-state) 3: Up-to-Down transition (i.e., end of the Up-state to beginning of the Down-state). A phase vector was calculated where 0 was the time point of transition 1, 50 was the time point of transition 2, and 100 was the time point of transition 3. The time points in between were filled with linearly spaced intermediate values, so that a phase vector was achieved with linear phase progression during the Down-state, and linear phase progression during the Up-state. All UDS were then divided into 40 bins per state (Gretenkord et al., 2017).

#### Calculation of Power at Higher Frequencies

Before calculating high frequency oscillatory power, the LFP data for each electrode channel was normalized to *z*-scores (Figure 1C). The normalized signal was then transformed using a continuous wavelet transform, specifically a complex Morlet wavelet for the theta (4–7.9 Hz), beta (15–29.9 Hz), gamma (30–79.9 Hz), and high-gamma (80–130 Hz) frequency bands (Massi et al., 2012; Gretenkord et al., 2017). The instantaneous area power was then calculated using trapezoidal numerical integration over all frequencies in a given band. The mean power per bin of the UDS phase vector was calculated for each UDS cycle (Figure 1D), leading to the alignment of the power to a normalized cycle (Valencia et al., 2013; Gretenkord et al., 2017). This compensated for the variable lengths of UDS cycles within a particular data segment, and allowed a mean power over the normalized UDS cycle to be calculated for each electrode channel, in each animal. From the plot showing the high-frequency oscillatory power distribution in the normalized SO cycle, we can determine the peak power (y) and the latency to peak power (x) (Figure 1D).

#### Spindle Detection

Our methods for spindle detection were adapted from Kim et al. (2015). Firstly, the spindle band-passed LFP (8–15 Hz) was normalized to the baseline and the instantaneous amplitudes of the analytical signal and of the Hilbert transform of the spindle band-passed signal were calculated. Secondly, the instantaneous amplitude was smoothed with a 40 ms kernel. The points of the smoothed signal that crossed the upper threshold (2.5 × the SD of the mean amplitude of the smoothed signal) were identified as potential spindles. Peaks in the spindle that were less than 200 ms apart were considered part of the same spindle. The crossing points of the lower threshold (1.5 × the mean amplitude of the smoothed signal) determined the duration of each spindle event. Finally, spindles that were shorter than 200 ms and longer than 2 s were rejected (Kim et al., 2015).

#### Sharp-Wave Ripple (SPW-R) Detection

For sharp-wave ripple (SPW-R) detection we also adapted the methods described by Kim et al. (2015). We used only recordings where the CA1 pyramidal layer could be confirmed *post hoc* by histology. The local LFP was band-passed for the ripple frequency range (80–130 Hz) and normalized to the baseline. The instantaneous amplitudes of the analytical signal and of the Hilbert transform of the ripple band-passed signal were calculated. The instantaneous amplitude was smoothed with a 15 ms kernel. The points of the smoothed signal that crossed the upper threshold (2 × the SD of the mean amplitude of the smoothed signal) were identified as potential ripples. Ripples with peaks less than 100 ms apart were considered to be one ripple event. The duration of the ripples was determined by the crossing points of the lower threshold (4 × SD of the mean amplitude of the smoothed signal). Finally, ripples that were shorter than 20 ms and longer than 400 ms were rejected.

#### Spindle and SPW-R Analysis

For spindles and SPW-Rs we measured the following; (1) the average amplitude of the event from zero to the peak of the LFP normalized to the baseline. (2) Duration of spindles/SPW-Rs determined as the time between the two points that crossed the lower threshold of the smoothed signal. (3) The variability over time (CV = SD/mean) of amplitude and duration. Other variables of interest were the spindle/SPW-R latency in the normalized SO cycle, the relative spindle/SPW-R density (number of events/Up-state), and the percentage of high-amplitude spindles (amplitude > 5 × mean event amplitude for each recording). Finally, the instantaneous frequency of each spindle/SPW-R event was calculated as the number of cycles of the spindle or SPW-R band-passed LFP that crossed the upper threshold of the smoothed signal, divided by time interval between the two crossing points.

### Neuronal Firing Analysis

Single neurons in the mPFC were first sorted online using the Plexon Sort Client and then offline using the Plexon Offline sorter. During offline sorting, all waveforms were aligned to the global minimum and short interspike intervals (ISIs) of (<1 ms) were automatically removed. Only neurons which could be well distinguished were included in the analysis. In most cases one neuron per channel was recorded, although occasionally two neurons could be isolated. No neurons were obtained from the hippocampus. The neuronal firing time point data were down-sampled to 1 kHz. Measures of coherence between the SO band-passed LFP and the neuronal firing were obtained using the mscohere function in MATLAB. The threshold firing rate for including a neuron for analysis was set at 10 spikes/min (0.17 Hz), while for coherence analysis this was set at 30 spikes/min (0.5 Hz). The magnitude-squared coherence was calculated as a function of the power spectral densities, *p*_*xx*_(*f*) and *p*_*yy*_(*f*) and the cross power spectral density, *P*_*xy*_(*f*), of x and y where x is the LFP and y the spike train of the neuron.

$$C_{xy}\left(f\right)=\frac{{\left|P_{xy}\left(f\right)\right|}^{2}}{p_{xx}\left(f\right)p_{yy}\left(f\right)}$$

The time points of neuronal firing were compared to the normalized Up-Down cycle to identify whether they occurred during an Up- or a Down-state. The percentage of firing on the Up- and Down-states was calculated as a percentage of the total firing. The neuronal firing frequency on the Up-state was calculated as a fraction of the total number of spikes occurring on the Up-states divided by the length of all the Up-states combined. The neuronal firing frequency on the Down-state was calculated in a similar manner. The neuronal firing frequency for the duration of the recording was determined by dividing the total number of spikes recorded, irrespective of state, by the duration of the recording. We also calculated the ISI and coefficient of variation (CV) of neuronal firing on the Up-state for the duration of the recording. Finally, we found the maximum coherence between the SO and neuronal firing on the Up-state and identified the frequency at which it occurred at (0.1 – 0.9 Hz).

### Statistical Analysis

Firstly, we ensured a Gaussian distribution of data and homogeneity of variance using the Kolmogorov–Smirnov and Levene’s Test respectively. If normality could not be confirmed, we performed a logarithmic or a square root transformation of the data. Specifically, we performed a logarithmic transformation for power on the Up- and Down-states and the mean peak power for all frequency bands in the mPFC and CA1. Mixed repeated measures ANOVA (RM ANOVA) analyses was conducted where mPFC subregion (four levels) was set as the within-subjects variable and the animal genotype as the between-subjects variable. A detailed comparison of the differences between mPFC subregions was out of the scope of the current study, however we proceeded with mPFC subregions as a repeated measure to identify any mixed effects/interactions with the genotype (Gretenkord et al., 2017). The outcome of the RM ANOVA analysis for the mPFC subregion is reported and, if the assumption of sphericity was violated, a Huynh-Feldt correction was applied. If a significant difference was found for genotype or a subregion/genotype interaction, follow-up univariate ANOVA analysis with a Bonferroni correction was performed for each mPFC subregion. Univariate ANOVA analysis was also performed for data obtained from the CA1 of the hippocampus as well as for neuronal data, as we combined neurons from all subregions of the mPFC. Non-parametric Mann–Whitney *U*-test with a Welch exact-correction analysis was performed for the SPW-R data where normal distribution could not be achieved. Parametric data is presented as mean ± standard error of the mean (SEM), while non-parametric data is presented as median ± interquartile range (IQR).

## Results

### SO Frequency Is Increased in the mPFC in A30P Mice

**FIGURE 2:**
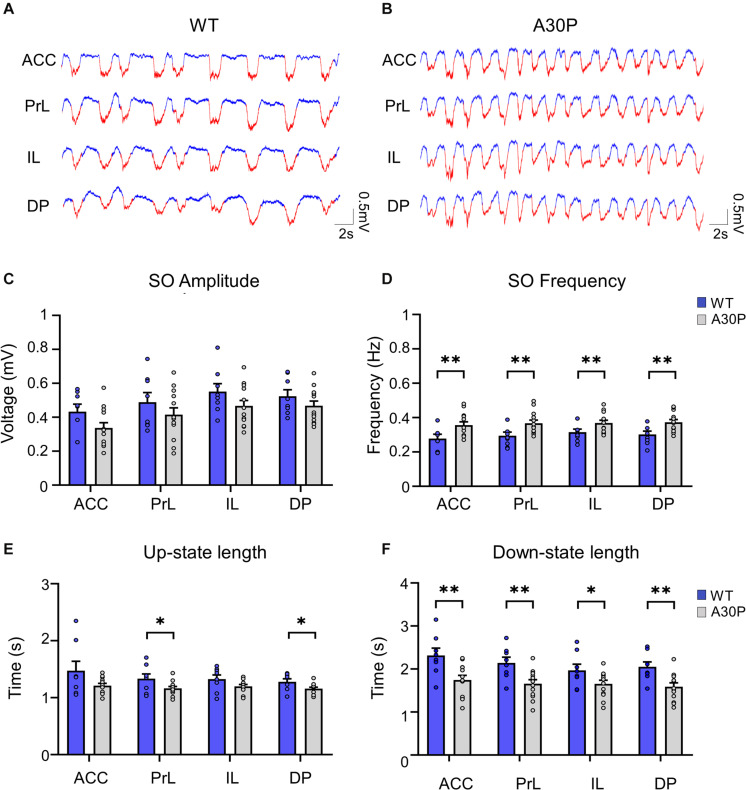
Changes in SO patterns in the mPFC of A30P animals. **(A,B)** Examples of Up- and Down-state detection (with red corresponding to the Up-state and blue to the Down-state) in the four sub-regions of the mPFC (ACC, PrL, IL, DP) of a WT and an A30P mouse. **(C)** The mean amplitude of the SO-band passed signal (0.1–0.9 Hz). **(D)** The SO frequency and **(E)** the mean length of the Up-state and **(F)** Down-state. Each variable is calculated as an average for each of the mPFC sub-regions (ACC, PrL, IL, DP) for A30P (*N* = 13) and WT (*N* = 8) animals. Error bars indicate the SEM and asterisks indicate significance: ^∗^*p* < 0.05, ^∗∗^*p* < 0.01.

Previous studies recording cortical activity in mice and rats using urethane anesthesia have reported sleep-state alternations between the SO (∼ 1 Hz) and a REM-like activity in which theta frequency activity predominates (Clement et al., 2008; Sakata and Harris, 2012; Pagliardini et al., 2013b). We also observed similar sleep-state changes but, at the dose of urethane used in the current study, we obtained ∼2–3 h periods of regular SO activity without long-lasting spontaneous alternations to REM-like activity (see section “Materials and Methods”). Regular SOs occurred simultaneously in all subregions of the mPFC in both A30P and WT mice aged 2.5–4 months (Figures 2A,B).

We first assessed whether α-syn pathology in A30P mice altered the properties of the SO. Although there was a trend for the SO to be smaller in all subregions of the mPFC in A30P mice there was no statistical difference in the amplitude of the SO (Figure 2C). However, the frequency of the SO was significantly faster in A30P compared to WT mice [RM ANOVA, *F*(1,19) = 9.64, *p* < 0.01], in all subregions of the mPFC (Figure 2D). A change in SO frequency could reflect shorter Up- and/or Down-states thus these were analyzed separately. We found that the Up-state was significantly shorter (Figure 2E) in the PrL and DP regions [*F*(1,19) = 5.93, *p* < 0.05], while the Down-state was significantly shorter in all mPFC regions [*F*(1,19) = 9.63, *p* < 0.01] in A30P mice (Figure 2F).

### Up-State Associated High-Frequency Oscillations in the mPFC

**FIGURE 3:**
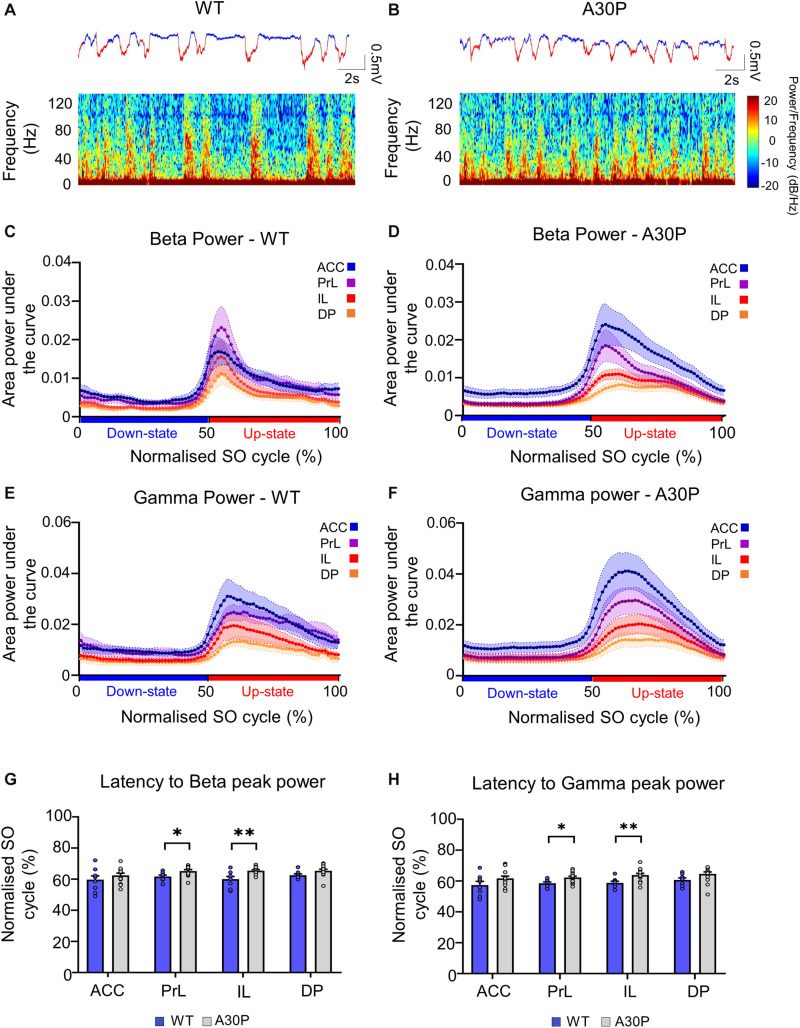
Changes in high-frequency oscillatory activity in the SO cycle in the mPFC of A30P mice. **(A,B)** Example LFP (red = Up-state and blue = Down-state) and corresponding spectrograms showing the power of oscillatory activity in the 0.1–130 Hz frequency spectrum, in the PrL region in **(A)** WT and **(B)** an A30P mouse. **(C)** The mean area power under the curve in the beta frequency range (15–29.9 Hz) in WT and **(D)** A30P mice. **(E)** The mean area power under the curve in the gamma frequency range (30–79.9 Hz) in WT and **(F)** A30P mice in the normalized SO cycle (0 = start of Down-state, 50 = Down to Up-state transition, 100 = end of Up-state). **(G)** The average latency to peak power in the normalized SO cycle in the beta and **(H)** gamma frequency ranges in WT (blue) and A30P (gray) mice. Each variable is calculated as an average for each of the mPFC sub-regions (ACC, PrL, IL, DP) for A30P (*N* = 13) and WT (*N* = 8) animals. Error bars indicate the SEM and asterisks indicate significance: ^∗^*p* < 0.05, ^∗∗^*p* < 0.01.

Fast network oscillations in the beta and gamma bands are associated with the Up-state of the SO (Steriade et al., 1996; Mukovski et al., 2007; Compte et al., 2008). We measured the average power content of the Up- and Down-state, the peak power and latency to peak power in the SO cycle in three frequency bands: beta (15–29.9 Hz), gamma (30–79.9 Hz) and high gamma (80–130 Hz) in the four mPFC subregions in WT and A30P mice (Figure 3).

We found that fast oscillatory activity was maximal over the early portion of the Up-state and then declined during the Up-state (Figures 3C–F). In addition, we observed that fast oscillations on the Up-state are larger in the more dorsal ACC and PrL regions. We compared activity in the beta, gamma and high-gamma bands in all four regions (Table 1). In WT mice the beta frequency activity on the Up-state occurred early in the normalized SO cycle, with a clear peak shortly after Up-state onset evident in all mPFC region (Figure 3C). In contrast the peak of the beta frequency activity in A30P mice was delayed relative to Up-state onset, and activity was distributed over a greater proportion of the Up-state, especially in ACC (Figure 3D). In WT mice gamma frequency band activity occurred more widely over the Up-state than for beta activity, although there was still a clear peak at the start of the Up-state (Figure 3E). In A30P mice gamma activity again appeared to peak later, and remained high for a larger proportion of the Up-state, particularly in ACC (Figure 3F). Although in A30P mice there was an apparent increase in area power for both the beta and gamma activity on the Up-state, particularly in ACC and PrL, this difference did not reach statistical significance (Table 1).

**TABLE 1:** Characterization of the high frequency oscillatory activity in the SO cycle, in the mPFC of WT and A30P animals.

	Band	Genotype	Region			
			ACC	PL	IL	DP
**Power on the Up-state (x10^3^)**	Beta	WT	9.28 ± 1.7	10.34 ± 1.39	7.64 ± 1.37	6.77 ± 1.19
		A30P	16.62 ± 3.51	12 ± 1.76	8.5 ± 1.22	6.98 ± 1.04
	Gamma	WT	21.50 ± 4.75	19.78 ± 2.72	15.76 ± 3.27	11.95 ± 2.29
		A30P	29.93 ± 5.95	23.52 ± 3.49	15.99 ± 3.09	12.49 ± 2.33
	High-Gamma	WT	10.11 ± 1.94	9.26 ± 1.17	7.41 ± 1.2	6.78 ± 1.24
		A30P	15.06 ± 3.33	11.19 ± 2.13	8.82 ± 2.2	7.9 ± 1.76
**Power on the Down-state (x10^3^)**	Beta	WT	4.52 ± 0.95	3.79 ± 0.84	3.32 ± 0.56	3 ± 0.52
		A30P	8.17 ± 1.87	4.65 ± 0.54	3.87 ± 0.54	3.77 ± 0.66
	Gamma	WT	8.29 ± 1.9	8.01 ± 1.9	6.41 ± 0.93	5.91 ± 1.41
		A30P	12.7 ± 2.55	8.56 ± 1.54	7.37 ± 1.68	7.02 ± 1.53
	High-Gamma	WT	6.53 ± 1.46	6.29 ± 1.26	5.71 ± 0.94	5.51 ± 1.14
		A30P	10.79 ± 2.62	8.1 ± 1.87	7.25 ± 1.99	6.92 ± 1.62
**Peak power (x10^3^)**	Beta	WT	55.78 ± 9.55	64.75 ± 9.39	47.21 ± 8.56	38.28 ± 6.89
		A30P	76.82 ± 18.93	58.24 ± 12.47	49.16 ± 13.56	42.38 ± 11.18
	Gamma	WT	93.12 ± 17.26	86.59 ± 11.89	65.00 ± 12.8	54.14 ± 9.29
		A30P	135.12 ± 28.76	102.92 ± 14.8	69.15 ± 11.42	53.48 ± 8.68
	High-Gamma	WT	58.5 ± 1.31	58.76 ± 0.93	57.35 ± 1.08	58.16 ± 0.98
		A30P	76.82 ± 18.93	58.24 ± 12.47	49.16 ± 13.56	42.38 ± 11.18
**Latency to peak power**	Beta	WT	57.38 ± 2.43	58.53 ± 0.84	58.77 ± 1.13	60.72 ± 1.33
		A30P	60.35 ± 1.28	61.48 ± 0.8	63.22 ± 0.94	64.28 ± 1.43
	Gamma	WT	67.17 ± 1.07	61.94 ± 1.02	60.41 ± 1.83	62.73 ± 0.86
		A30P	62.49 ± 1.41	65.2 ± 0.92	65.46 ± 0.68	65.39 ± 1.02
	High-Gamma	WT	51.63 ± 11.56	48.9 ± 8.66	40.01 ± 8.6	36.85 ± 8.42
		A30P	58.25 ± 0.76	58.88 ± 0.94	57.21 ± 0.99	56.37 ± 1.16

*The average of each variable ± SEM are shown for three frequency bands (Beta, Gamma, High-Gamma), in the mPFC subregions (ACC, PrL, IL, DP), in WT (N = 8) and A30P (N = 13) mice.*

However, significant differences between A30P and WT animals were found in the latency to peak beta [Figure 3G; *F*(1,19) = 6.36, *p* < 0.05] and gamma [Figure 3H; *F*(1,19) = 6.81, *p* < 0.05, RM ANOVA] power in the PrL and IL regions. The normalized SO cycle consists of the Down-state followed by the Up-state, where 0% in the normalized SO cycle is the beginning of the Down-state, 50% is the Down-to-Up-transition and 100% is the Up-state termination (see section “Materials and Methods”). In the PrL region the beta power peak latency was 61.5% ± 0.8% in A30P animals compared to 58.5 ± 0.84% in WT animals. In the IL subregion the beta power peak latency was 63.2 ± 0.94% in A30P compared to 58.9 ± 1.0% in WT animals. We did not find any significant differences in the high-gamma frequency band between WT and A30P mice (Table 1). Thus overall in the young A30P mice, although Up-state associated fast oscillations did not differ in magnitude from WT activity, there was a clear delay in the latency to peak power on the Up-state for both the beta and gamma band activity.

### Sleep Spindles in the mPFC Are Altered in A30P Mice

**FIGURE 4:**
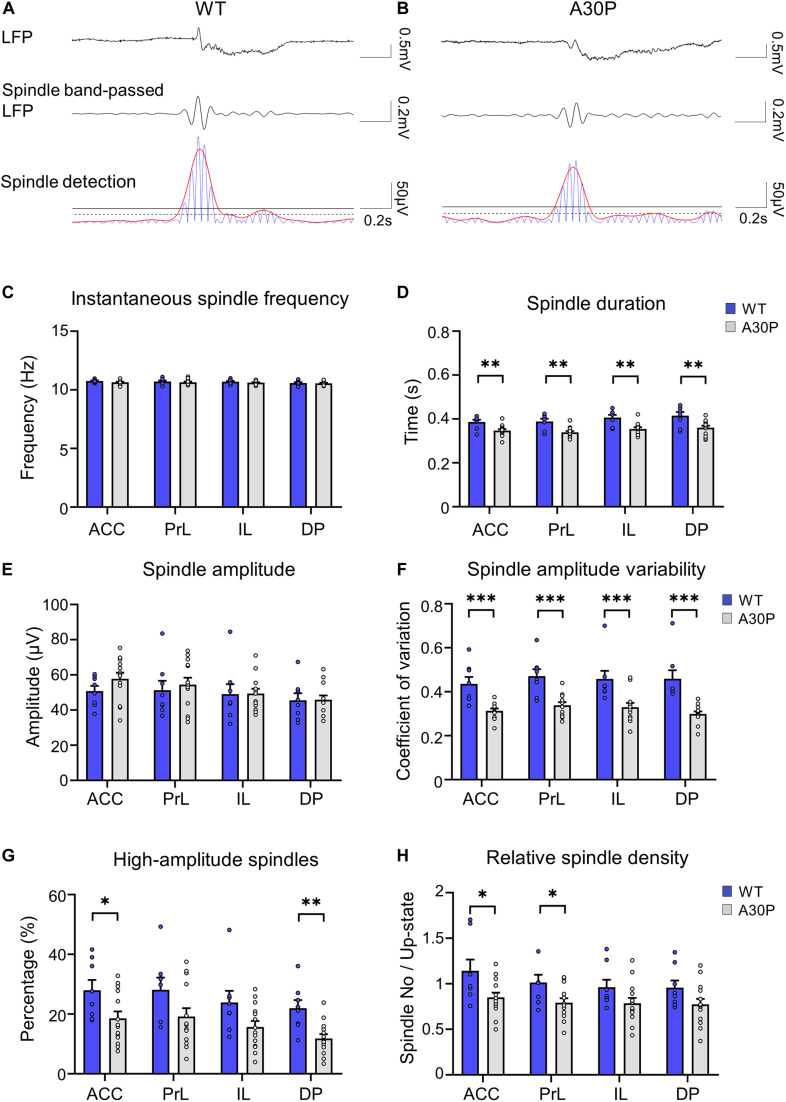
Changes in sleep spindle characteristics in the mPFC of A30P mice. **(A,B)** Examples of spindles in the LFP, the spindle-band passed LFP and the spindle detection signal in the ACC of a WT and an A30P mouse. The spindle properties are characterized by **(C)** the average instantaneous spindle frequency, **(D)** spindle duration, **(E)** spindle amplitude, **(F)** spindle amplitude variability (CV), and **(G)** percentage of high-amplitude spindles. **(H)** The relative spindle density indicates the average number of spindles per Up-state number. Each variable was extracted for the four subregions of the mPFC (ACC, PrL, IL, DP) in WT (*N* = 8, blue) and A30P (*N* = 13, gray) animals. Error bars indicate the SEM and asterisks indicate significance: ^∗^*p* < 0.05, ^∗∗^*p* < 0.01, ^***^p < 0.001.

In view of the early changes in spindles reported in patients with PD and DLB (Christensen et al., 2014; Latreille et al., 2015; Fernández-Arcos et al., 2019), we were interested to see if changes in spindles occurred as a consequence of the α-syn pathology in the young A30P mice. Spindles with a duration > 0.3 s, were observed in the LFP in both WT and A30P mice (Figures 4A,B) in all subregions of the mPFC. As can be seen in the examples (Figures 4A,B), spindles predominantly occurred at the beginning of the Up-state cycle in both WT and A30P mice. Averaged across all subregions in the mPFC spindle frequencies were not significantly different between WT and A30P mice at 10.65 ± 0.04 Hz and 10.7 ± 0.06 Hz, respectively (Figure 4C).

However, we did find that the spindle duration was significantly shorter in A30P mice compared to WT mice [*F*(1,19) = 14.21, *p* < 0.01], in all mPFC subregions (Figure 4D). In addition, although there was no significant change in spindle amplitude between WT and A30P mice (Figure 4E), spindle amplitude CV was significantly lower in A30P animals compared to WT animals [*F*(1,19) = 22.39, *p* < 0.01] in all mPFC subregions (Figure 4F). These findings could suggest that A30P mice have less high-amplitude spindles compared to WT mice so we measured the percentage of high-amplitude spindles (see section “Materials and Methods”). This revealed that in A30P animals high-amplitude spindles made up a significantly lower percentage of the total spindles [*F*(1,19) = 6.17, *p* < 0.05] in all subregions of the mPFC (Figure 4G). Finally, as the increase in SO frequency, outlined above, results in more Up-states in A30P mice we compared the relative spindle density (number of spindles per Up-state), and found a significant decrease in A30P mice [*F*(1,19) = 4.71, *p* < 0.05] in the ACC and PrL regions, with a similar trend in the other subregions (Figure 4H). Overall, therefore, although spindle frequency did not differ between WT and A30P mice, the duration, relative density and number of high-amplitude spindles were all significantly reduced in the young A30P mice suggesting marked impairment in spindle generation.

### SO Frequency Is Increased in the Hippocampus in A30P Mice

**FIGURE 5:**
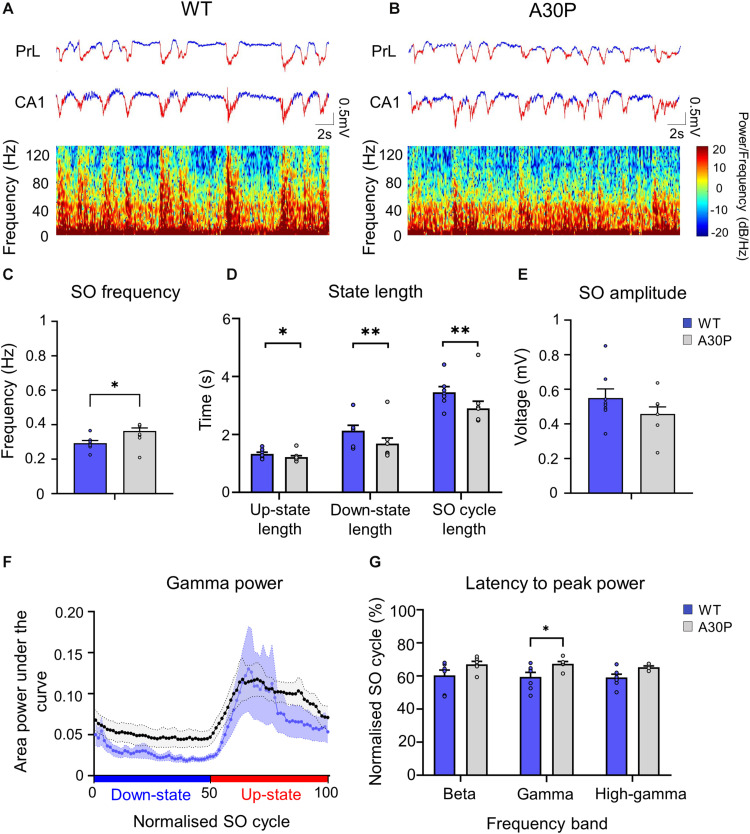
Changes in SO patterns in the CA1 of the hippocampus of A30P mice. **(A,B)** Example of Up- and Down-state detection (red = Up-state and blue = Down-state) in the LFP recorded simultaneously from the PrL region of the mPFC and from the ipsilateral CA1 region of the hippocampus, in a WT and an A30P mouse. The spectrograms show the distribution of oscillatory activity power (0.1–130 Hz) in the CA1 region of the hippocampus in relation to Up- and Down-states. **(C)** The SO frequency, **(D)** the mean length of the Up-state, Down-state and SO cycle and **(E)** the mean SO amplitude in the CA1 region of the hippocampus are shown for WT (*N* = 7; blue) and A30P (*N* = 8; gray) mice. **(F)** Changes in the distribution of power in the gamma frequency band (30–79.9 Hz) in the normalized SO cycle (0 = start of Down-state, 50 = Down to Up-state transition,100 = end of Up-state), in the CA1 region of the hippocampus in WT and A30P mice. **(G)** The mean latency to peak power in the beta (15–30 Hz), gamma (30–80 Hz) and high-gamma (80–130 Hz) frequency ranges in the normalized SO cycle. All power-related variables **(F,G)** indicate the mean across WT (*N* = 7; blue) and A30P (*N* = 7; gray) animals. Error bars indicate the SEM and asterisks indicate statistical significance: ^∗^*p* < 0.05, ^∗∗^*p* < 0.01.

A SO was also observed in CA1 of hippocampus (Figure 5) and occurred concurrently in mPFC and hippocampus in both WT and A30P mice (Figures 5A,B). As reported above for mPFC the hippocampal SO was also significantly faster (Figure 5C) in A30P compared to WT mice [*F*(1,13) = 17.57, *p* < 0.01]. Also consistent with the mPFC findings, the increased SO frequency in A30P animals was due to a shorter SO cycle length [*F*(1,13) = 14.54, *p* < 0.01] composed of both shorter Up- [*F*(1,13) = 6.49, *p* < 0.05] and Down-states [*F*(1,13) = 10.19, *p* < 0.01] (Figure 5D). However, the SO amplitude did not differ between WT and A30P mice (Figure 5E).

### The Distribution of Up-State Associated High-Frequency Activity Is Altered in A30P Mice in the Hippocampus

Fast network oscillations are nested on the Up-state of the hippocampal SO (Figure 5) but the gamma frequency activity was not as well aligned to the start of the Up-state as seen in the mPFC (compare Figure 5F with Figure 3D). In WT mice the gamma activity power was prominent over the first half of the Up-state but then declined toward the end of the Up-state. In A30P mice the peak gamma power appeared broader and remained high over a greater portion of the Up-state. Although there was no difference in the power of the gamma activity between WT and A30P mice the latency to peak gamma power in the normalized SO cycle was longer in A30P mice [*F*(1,13) = 5.48, *p* < 0.05, Univariate ANOVA], but there was no change in the latency to peak beta or high-gamma power (Figure 5G). Thus, similar to our observations in the mPFC, there was a delay in the occurrence of the gamma oscillation peak power on the Up-state in the hippocampus, but we did not find any difference in the average power of the fast oscillatory activity in any frequency band on the Up- or Down-states (Table 2).

**TABLE 2:** Characterization of the high frequency oscillatory power in the SO cycle in CA1 of the hippocampus of WT and A30P animals.

Variable	Band	WT	A30P
Power on the Up-state (×10^3^)	Beta	40.86 ± 12.5	68.21 ± 8.42
	Gamma	76.8 ± 25.55	95.21 ± 14.95
	High-gamma	18.26 ± 5.28	24.49 ± 4.64
Power on the Down-state (×10^3^)	Beta	17.45 ± 4.07	31.8 ± 6.03
	Gamma	27.5 ± 6.21	49.69 ± 12.03
	High-gamma	7.2 ± 1.78	16.47 ± 3.31
Peak power (×10^3^)	Beta	269.56 ± 115.57	292.02 ± 34.24
	Gamma	494.7 ± 211.99	445.33 ± 68.38
	High-gamma	129.26 ± 31.43	150.1 ± 17.87
Latency to peak Power (%)	Beta	60.36 ± 3.26	66.34 ± 1.68
	Gamma	59.47 ± 2.77	66.71 ± 1.38
	High-gamma	59.12 ± 1.79	63.9 ± 1.52

*The average of each variable ± SEM is shown for three frequency bands (beta, gamma, high-gamma), for the WT (N = 7) and A30P (N = 8) animal groups.*

### A30P Mice Have Normal Sharp Wave Ripples (SPW-R) in the Hippocampal CA1 Region

In view of the changes outlined above in spindles seen in A30P mice we went on to assess the impact of abnormal α-syn on the generation of ripples in the hippocampus, which are temporally correlated with cortical spindles (Siapas and Wilson, 1998). Ripples had an instantaneous frequency of 87.48 ± 6.46 Hz in WT and 83.43 ± 1.73 Hz in A30P animals and were not significantly different (Table 3). Moreover, ripples were similarly located at the beginning of the SO cycle with a latency of 59.58 ± 8.3% Hz in WT and 61.84 ± 6.45% in A30P animals. Overall, we found no significant differences in ripples between WT and A30P mice in any of the variables tested, including the instantaneous ripple frequency, ripple duration and amplitude, and variability over time (CV). Furthermore, the relative SPW-R density (number of SPW-Rs/Up-state, and the total number SPW-Rs), were also not significantly different between genotypes (Table 3).

**TABLE 3:** SPW-R characterization in the CA1 of the hippocampus of WT and A30P animals.

Variable	WT	A30P
Duration (ms)	194.8 ± 74.37	160.64 ± 32.47
Duration CV	0.45 ± 0.14	0.55 ± 0.07
Amplitude (μV)	34.63 ± 16.13	28.34 ± 12.45
Amplitude CV	0.33 ± 0.19	0.34 ± 0.11
Density–Number/Up-state (×10^3^)	1.13 ± 0.91	1.24 ± 5.48
Peak latency in the SO cycle (%)	59.58 ± 8.3	61.84 ± 6.45
Instantaneous frequency (Hz)	87.48 ± 6.46	83.43 ± 1.73

### A30P Mice Have Altered Neuronal Firing Patterns in Relation to the SO

**FIGURE 6:**
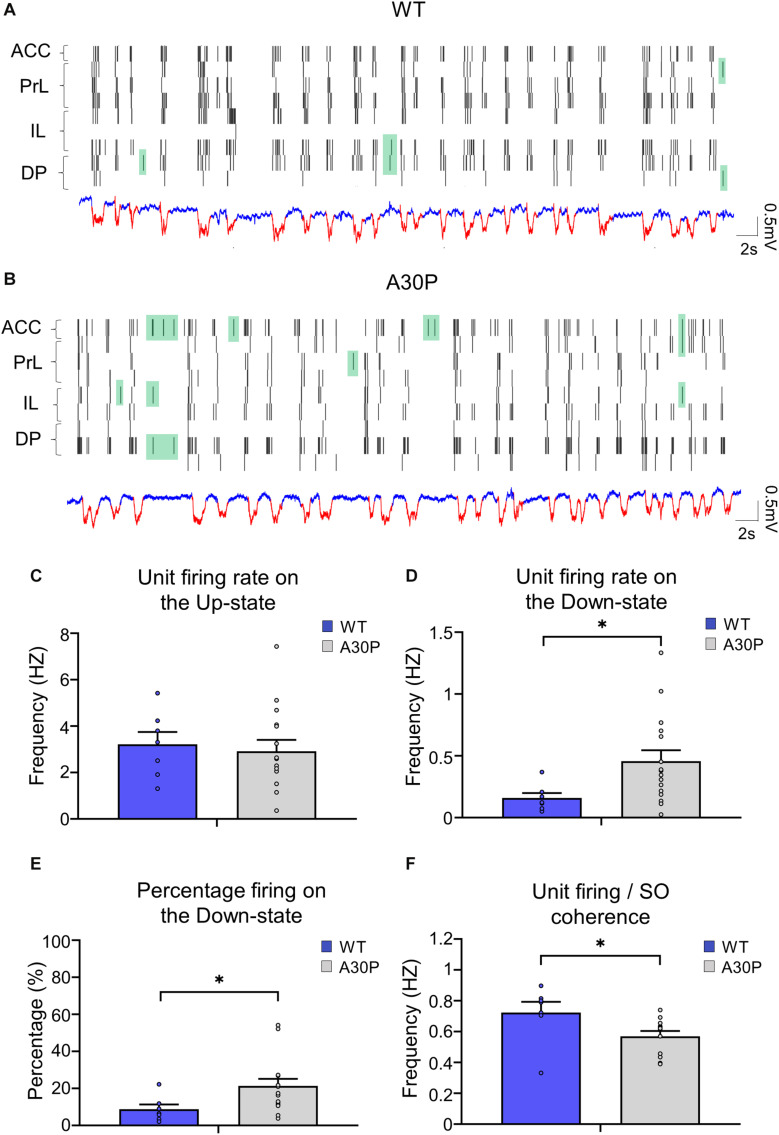
Changes in neuronal firing patterns in relation to the SO in the mPFC of A30P mice. **(A,B)** Example raster plots of the neuronal firing in the sub-regions of the mPFC (ACC, PrL, IL, DP), in relation to Up- and Down-states detected in a PrL channel in a WT and an A30P mouse (red = Up-state and blue = Down-state). Spikes that occur on the Down-state are indicated in green. The neuronal firing frequency on **(C)** the Up- and **(D)** the Down-states, and **(E)** the percentage of the total firing occurring on the Down-state were averaged across mPFC subregions: ACC (WT = 5/7, A30P = 9/13), PrL (WT = 3/6, A30P = 8/16), IL (WT = 4/5, A30P = 7/13) and DP (WT = 4/5, A30P = 7/9), for WT (*N* = 8, blue) and A30P (*N* = 15, gray) animals. **(F)** The average neuronal firing/SO coherence across all neurons recorded from each animal (WT = 8, A30P = 12) is also shown for all mPFC regions combined: ACC (WT = 5/7, A30P = 10/15), PrL (WT = 3/6, A30P = 9/19), IL (WT = 4/6, A30P = 9/19) and DP (WT = 5/7, A30P = 6/12) where numbers in brackets indicate the number of animals/number of neurons. Error bars indicate the SEM and asterisks indicate statistical significance: ^∗^*p* < 0.05.

As the changes in SO and Up-state associated oscillatory activity in the mPFC outlined above could reflect changes in neuronal firing in A30P mice we compared neuronal properties in WT and A30P mice (Figure 6). Neurons were recorded in the four subregions of the mPFC and data were combined. Overall neuronal firing frequency on the Up-state (Figure 6C; WT = 2.76 ± 1.47 Hz; A30P = 3.39 ± 2.71 Hz) was not significantly different between WT and A30P mice. However, in A30P mice we observed more firing on the Down-state (Figure 6D). Overall the firing frequency on the Down-state was significantly increased from 0.16 ± 0.04 Hz in WT mice, to 0.47 ± 0.09 Hz in A30P mice [*F*(1,20) = 4.89, *p* < 0.05]. Consistent with this the percentage of the total firing occurring on the Down-state (Figure 6E) in A30P mice (19.67 ± 3.38%) was also significantly higher than in WT animals [10.08 ± 2.16%; *F*(1,20) = 4.75, *p* < 0.05]. The neuronal firing/SO coherence (Figure 6F) was also significantly lower in A30P (0.68 ± 0.06) compared to WT animals [0.57 ± 0.05; *F*(1,17) = 4.95, *p* < 0.05]. Our findings indicate increased in neuronal firing during the predominantly “silent” Down-state, suggesting aberrant neuronal activity.

## Discussion

We have identified a number of significant changes in the SO in both the mPFC and CA1 of the hippocampus of A30P mice under urethane anesthesia as a consequence of the expression of human mutant α-syn. The SO frequency was faster in A30P mice in both the mPFC and hippocampus as a result of the shorter Up- and Down-states. In addition, the distribution of power on the Up-state was altered in A30P mice, with an increased latency to peak power in the beta and gamma frequency bands in the mPFC and gamma band in hippocampus. We also found that in A30P mice a greater percentage of the total firing occurred on the Down-state, and there was a lower neuronal firing/SO coherence compared to WT mice. There were no changes in the total power of beta, gamma or high-gamma activity on the Up-state between A30P and WT animals in either the mPFC or hippocampus, and no change in hippocampal ripples. However, interestingly, A30P mice had impaired spindle activity, including reduced spindle duration, less high-amplitude spindles and lower relative spindle density. These early changes in sleep-related oscillations in A30P mice are occurring before any reported cognitive or motor symptoms which only emerge from ∼12 months of age (Freichel et al., 2007; Schell et al., 2009), although it is possible further behavioral testing in young A30P mice might reveal deficits in different cognitive tasks.

### SO Frequency Is Faster in A30P Mice

A key finding of the current study was an increase in SO frequency recorded under urethane anesthesia in both mPFC and hippocampus in A30P animals. Despite the known functional and anatomical differences between the mPFC subregions (Kesner and Churchwell, 2011), the increase in SO frequency occurred in all mPFC subregions. At 2.5–4 months of age, as used in the current study, mild pathology is present in A30P mice but no cognitive deficits have been reported (Freichel et al., 2007; Schell et al., 2009), so the change in SO represents an early network dysfunction due to α-syn pathology. Although the function of α-syn is still being explored, it is a synaptic protein involved in the regulation of neurotransmitter vesicle release (Lautenschläger et al., 2017). Therefore, subtle early changes in either excitatory or inhibitory neuronal function could lead to an increase in excitability within the cortical and hippocampal networks that impacted SO frequency.

In contrast to our findings in A30P mice, studies in both the rTg4510 model of tauopathy (Menkes-Caspi et al., 2015), and the 3xTg-AD model (Castano-Prat et al., 2019), have reported a slowing of the cortical SO frequency. The SO was slower in both awake-behaving conditions and under anesthesia in the model of pathological tau (Menkes-Caspi et al., 2015), suggesting that the changes in SO frequency are not solely due to effects of anesthesia. However, one study in an AD model (APP23 × PS45) did report a significant increase in SO frequency, that could be reversed following cortical surface application of midazolam to increase GABA_*A*_ receptor-mediated neurotransmission (Busche et al., 2015). The reason for the different changes in SO frequency are not clear because, in all the AD murine models studied, experiments were performed at an age when the Tg mouse models exhibited significant pathology and cognitive deficits (Busche et al., 2015; Menkes-Caspi et al., 2015; Castano-Prat et al., 2019). However, the discrepancy may still relate to disease stage as, although SO frequency was slowed at 20 months of age in the 3xTg-AD model, there was a non-significant trend for an increase in SO frequency at 7 months of age (Castano-Prat et al., 2019). It would be interesting to assess, in the equivalent of the prodromal disease stage before cognitive dysfunction is reported, whether there are increases in SO frequency in these other models of neurodegeneration as we have seen here in A30P mice. Future studies in aged A30P mice are also required in order to determine whether a later slowing of the SO occurs as a result of increased α-syn pathology as the disease progresses. As well as the SO changes in mPFC, we also saw a similar increase in SO frequency in CA1 of the hippocampus. Hippocampal and cortical SOs can arise from different local circuits, although there is a close association between the two regions (Wolansky et al., 2006). Therefore, the increased SO frequency we observed in the hippocampus in A30P mice could reflect either local circuit changes due to α-syn pathology and/or indirect changes mediated via strong cortical inputs from the mPFC (Thierry et al., 2000).

Interestingly, in recent studies cortical optogenetic stimulation at normal SO frequencies in a mouse model of AD restored SO power and slowed down amyloid-β pathology (Kastanenka et al., 2017), while artificially increasing SO frequency from 0.6 to 1.2 Hz exacerbated AD pathology and intracellular Ca^2+^ overload (Kastanenka et al., 2019). Thus it is possible that in young A30P mice, the increased SO frequency we have observed could reflect an early network hyperexcitability that might further exacerbate α-syn aggregation, resulting in faster progression of neurodegeneration.

### Mechanisms Underlying the Increase in SO Frequency in A30P Mice

A complex interplay of different mechanisms regulates Up- and Down-state transitions including, K^+^ conductances and excitatory and inhibitory inputs (Haider et al., 2006; Sanchez-Vives, 2020) and parvalbumin (PV+)- and somatostatin (SST +)-expressing interneuron firing (Zucca et al., 2017). Recently it was shown that the firing rate during the Down-state was inversely correlated with the Down-state length, so higher firing rates during the Down-state led to a shorter Down-state, and thus faster SO frequency (Castano-Prat et al., 2019). Therefore, in A30P mice the higher firing rate on the Down-state may directly contribute to the faster SO frequency. Another finding of our study was a decrease in Up-state duration in A30P mice. The Up-state is also governed by a tight excitation and inhibition balance, specifically NMDAR-mediated glutamatergic activity and GABAergic inhibition (Haider et al., 2006). Reduced GABA_*A*_ receptor function can shorten Up-state duration (Sanchez-Vives and McCormick, 2000) so abnormal firing of interneurons on the Up-state could potentially lead to shorter Up-states in A30P mice. Both PV+ and SST+ interneurons fire on the Up-state and SST+ cells have been shown to be involved in synchronous Up-state termination (Zucca et al., 2017). Further studies are needed to make a detailed assessment of possible α-syn-related changes to the different interneuron populations in A30P mice that could contribute to the shorter Up- and Down-states and faster SO frequency.

### The Distribution of High Frequency Oscillations on the Up-State Is Altered in A30P Mice

As shown previously (Valencia et al., 2013; Gretenkord et al., 2017), we found that fast oscillatory activity was maximal over the early portion of the Up-state and then declined during the Up-state. In addition, as we observed in rat (Gretenkord et al., 2017), fast oscillations on the Up-state are larger in the more dorsal ACC and PrL regions. Greater fast oscillatory network activity over the early portion of the Up-state is consistent with neuronal temporal firing patterns in which interneurons fire at the start of the Up-state, while pyramidal cells tend to fire more uniformly across the Up-state (Luczak and Barthó, 2012). However, in A30P mice there was an increased latency to peak power of the beta and gamma activity on the Up-state in most regions. Although we did not see an overall change in the amount of neuronal firing activity on the Up-state in A30P mice, it is possible that neuronal firing occurs in a more variable manner on the Up-state. Such a change in spike timing could account for both the increase in peak latency, and the broader spread of activity over the duration of the Up-state that we observed in A30P mice.

### A30P Animals Have Reduced Spindle Activity

In this study, spindles predominantly occurred at the start of the Up-state as shown previously (Barthó et al., 2014), and the frequency of spindle activity (∼9–11 Hz) was similar to other rodent studies conducted recording under urethane anesthesia (Barthó et al., 2014). Interestingly, however, we found clear evidence of a disruption in spindle frequency activity in young A30P mice, including less high-amplitude spindles and a lower relative spindle density. Spindle duration was also shorter in A30P mice compared to WT mice, although the spindle frequency was similar. Spindles are generated in the thalamus during slow wave sleep through an interplay between GABAergic thalamic reticular nucleus (TRN) neurons and thalamocortical (TC) relay cells (Steriade et al., 1987; Gardner et al., 2013). Therefore, our observation of a shorter spindle duration could be attributed to either altered engagement of TRN neurons in the spindle event and/or changes in TC neurons. In addition to the neocortex and hippocampus, α-syn pathology is also present in thalamic structures in A30P mice (Neumann et al., 2002), but the impact of abnormal α-syn on the function of this region is unknown. Overall, our data showing impaired spindles in A30P mice, suggests a disruption to thalamo-cortical circuits, which would be consistent with the proposed role of the thalamus in some of the key clinical features of DLB such as the cognitive fluctuations and visual hallucinations (Delli Pizzi et al., 2015).

Spindle impairments have been observed in DLB, PDD and PD patients (Christensen et al., 2014; Latreille et al., 2015). A polysomnographic study found that some patients with DLB had no sleep spindles, while others had lower-frequency sleep spindles (Fernández-Arcos et al., 2019). A similar decrease in spindle density was found in PD patients (Christensen et al., 2014). Spindle changes were also present early in the disease stage as PD patients who progressed to dementia had significantly decreased spindle density, and lower spindle amplitude in posterior cortical areas (Latreille et al., 2015). Studies in prodromal DLB are needed to determine whether spindle deficits also occur and could be a potential early biomarker of DLB.

### Functional Consequences of α-syn Mediated SO and Spindle Dysfunction

The increase in SO frequency we have seen in A30P mice could have a negative impact on the important functions of sleep. The Up-state provides a temporal framework for neurons in the hippocampus and cortex to communicate with each other and bind into the functional assemblies that underlie memory consolidation (Timofeev and Chauvette, 2017; Klinzing et al., 2019). This hippocampal-cortical communication enables the declarative memories acquired when awake to become increasingly cortically dependent. On the other hand, Down-states are crucial for synaptic strength homeostasis (Watson et al., 2016; Timofeev and Chauvette, 2017).

The animals used in this study represent a prodromal model of DLB, where no measurable cognitive dysfunction has yet been reported, so we would not expect widespread and dramatic changes in sleep-related oscillations as this stage in A30P mice. Thus, for example we did not find any changes in hippocampal ripple oscillations, similar to a recent study in a murine model of AD (Zhurakovskaya et al., 2019), although others do report SPW-R deficits in murine models of dementia (Witton et al., 2016), and AD (Iaccarino et al., 2016; Benthem et al., 2020). However, the early deficits in sleep spindles in A30P could be an indicator of network abnormalities that will ultimately lead to progressive memory dysfunction.

Abnormal slow wave sleep activity is thought to prevent the clearance of pathological forms of amyloid-β and tau, and even promote excess production of these peptides (Mander et al., 2016). If the same were also true for α-syn clearance, then the sleep oscillation impairments we have observed in A30P mice could set off a cycle of increasing levels of α-syn and sleep disruption, as has been suggested might occur in PD (Bohnen and Hu, 2019).

### The Clinical Significance, Future Directions and Limitations

The early changes seen in our study are important for understanding the impact of abnormal α-syn pathology on network oscillations. Future work is needed to confirm that the findings reported here are also observed in A30P mice during natural sleep. In addition, it would be important to assess how changes in sleep-related activity alter with increasing age and disease progression in A30P mice as we have previously shown an age-dependent deficit in gamma frequency activity *in vitro* in A30P mice (Robson et al., 2018). It would also be interesting to determine whether interventions aimed at, for example, boosting spindles would delay the onset of cognitive decline in A30P mice.

Hyperexcitability and epilepsy are well described in AD patients (Born, 2015; Vossel et al., 2016, 2017) and thus, antiepileptic medications have been considered potentially beneficial for these patients (Pandis and Scarmeas, 2012). In view of our findings, which could suggest early network hyperexcitability in A30P mice, as well as the evidence of a high incidence of myoclonus (a form of cortical hyperexcitability) in patients with DLB (Morris et al., 2015; Beagle et al., 2017), antiepileptic drugs such as levetiracetam could be beneficial in patients with DLB. Myoclonus can also be treated with levetiracetam (Caviness, 2014), thus it would be interesting to see if drugs aimed at reducing hyperexcitability could restore the changes in sleep-related oscillations we have observed as a result of the α-syn pathology in the A30P mouse model. Interestingly, epilepsy and myoclonus have recently been reported in A53T α-syn mice which was associated with reduced NREM sleep and more time spent in the awake state (Peters et al., 2020). Ours and other data, therefore, support the idea that early interventions aimed at improving sleep cycle regulation and sleep-related oscillations could be beneficial, not only in AD (Mander et al., 2016) and PD (Bohnen and Hu, 2019), but also in patients with DLB.

## Data Availability Statement

The raw data supporting the conclusions of this article will be made available by the authors, without undue reservation.

## Ethics Statement

The animal study was reviewed and approved by the UK Home Office PIL.

## Author Contributions

MS: investigation and visualization. MS and BZ: formal analysis. MS, BZ, AT, J-PT, and FL: editing and revising the manuscript and reviewing and editing. J-PT, AT, and FL: supervision and funding acquisition. MS and FL: conceptualization. FL: writing. All authors contributed to the article and approved the submitted version.

## Conflict of Interest

The authors declare that the research was conducted in the absence of any commercial or financial relationships that could be construed as a potential conflict of interest.
